# The anti-obesity effects of a water-soluble glucan from *Grifola frondosa via* the modulation of chronic inflammation

**DOI:** 10.3389/fimmu.2022.962341

**Published:** 2022-07-28

**Authors:** Xue Jiang, Jie Hao, Yanfeng Zhu, Zijian Liu, Lanzhou Li, Yulin Zhou, Yu Li, Lirong Teng, Di Wang

**Affiliations:** ^1^ School of Life Sciences, Jilin University, Changchun, China; ^2^ Engineering Research Center of Chinese Ministry of Education for Edible and Medicinal Fungi, Jilin Agricultural University, Changchun, China

**Keywords:** polysaccharides, *grifola frondosa*, structural analysis, chronic inflammation, diet-induced obesity

## Abstract

Polysaccharides from *Grifola frondosa* (*G. frondosa*) have anti-obesity and anti-inflammatory activities. In this study, the major type, molecular weight, homogeneity and structure of a polysaccharide purified from *G. frondosa* (denoted GFPA) were determined. In high-fat diet (HFD)-treated mice, 8 weeks of GFPA administration efficiently decreased body weight and blood glucose concentration and counteracted hyperlipidemia. GFPA efficiently decreased adipocyte size and ameliorated inflammatory infiltration in the three types of white adipose tissue and alleviated steatosis, fat accumulation and inflammatory infiltration in the livers of HFD-fed mice. GFPA also decreased the concentrations of aspartate aminotransferase, alanine aminotransferase and pro-inflammatory factors in the sera and livers of HFD-treated mice. Furthermore, GFPA was found to regulate lipid metabolism *via* the inhibition of ceramide levels in HFD-treated mice. GFPA exhibited strong anti-obesity effects *via* the modulation of chronic inflammation through Toll-like receptor 4/nuclear factor kappa-B signaling, which supports the use of GFPA for the treatment of obesity.

## 1 Introduction

Obesity is characterized by excessive weight gain and fat deposition. It has become a major threat to human health globally, affecting 51% of the world’s population (1). Hyperlipidemia, chronic low-grade inflammation and insulin resistance, which occur concurrently with obesity, may lead to non-alcoholic fatty liver disease, cardiovascular disease and diabetes mellitus (2).

The link between chronic inflammation and obesity has been widely researched (3, 4). Chronic inflammation, which is driven by a series of adverse dietary factors, is a characteristic of obesity-related metabolic disorders (5). Accordingly, a practicable strategy for the clinical treatment of obesity, by reducing ceramide (Cer) levels and chronic inflammation induced by overfeeding, has been reported (6). The activation of Toll-like receptors by endogenous by-products of metabolism or exogenous agents promotes chronic inflammation, leading to the activation of nuclear factor kappa-B (NF-κB) and the production of inflammatory cytokines (7, 8).

Most patients with obesity show poor compliance with lifelong behavioral therapies, such as a balanced diet and physical exercise (9). The surgical procedures for weight loss in obese patients are mainly based on malabsorptive or restrictive approaches, which have a high cost and lead to various complications, including perioperative death (10). Although lipid-lowering drugs (fibrates, acipimox, lovastatin and simvastatin (SV)) have modest clinical efficacy, their potential adverse effects, including rhabdomyolysis and hepatotoxicity, are not negligible (11). Natural products have been reported to prevent hyperlipidemia and regulate chronic inflammation, and therefore, they are candidate agents for obesity treatment, with lower costs and fewer adverse effects than lipid-lowering drugs (12).

Polysaccharides isolated from mushrooms mainly contain a β-linked glucose (Glc) backbone, with different patterns and degrees of branching in various species. These polysaccharides have anti-obesity activity (13). The polysaccharide obtained from the sporoderm-broken spores of *Ganoderma lucidum*, which is a β-D-glucan containing (1→3)-β-D-Glcp, (1→3,6)-β-D-Glcp, (1→6)-β-D-Glcp and terminal-β-D-Glcp moieties, suppresses obesity and inflammation by inhibiting Toll-like receptor 4 (TLR4)/myeloid differential protein-88 (Myd88)/NF-κB signaling in mice fed a high-fat diet (HFD) (2). *Dictyophora indusiata* polysaccharides, which contain Glc, mannose and galactose (Gal), have anti-obesogenic and anti-inflammatory effects through the regulation of the intestinal microbiome and inflammatory cascades in HFD-fed mice (14).

We have previously shown that *Grifola frondosa* (*G. frondosa*), which belongs to the Polyporaceae family, regulates lipid metabolism by inhibiting Cer, which further contributes to its anti-obesity effects in mice with diet-induced obesity (DIO) (15). As one of the main bioactive substances of *G. frondosa*, crude aqueous polysaccharide extracts have been shown to ameliorate lipid metabolic disorders in HFD-fed rats (16). One of the polysaccharides purified from *G. frondosa* prevents lipopolysaccharide/D-galactosamine-induced acute liver injury (17). However, the structure of polysaccharides purified from *G. frondosa* and their anti-obesity effects through the modulation of chronic inflammation have not been systemically reported.

In this study, we performed the structural characterization of a polysaccharide purified from *G. frondosa* (termed GFPA). The anti-obesity effects of GFPA were found to be related to the inhibition of Cer, *via* the downregulation of TLR4-mediated pro-inflammatory signaling cascades in mice with DIO. These results support the use of GFPA as an anti-obesity agent that targets chronic inflammation.

## 2 Materials and methods

### 2.1 Purification of GFPA

*G. frondosa* fruiting bodies (Gutian Tianxian Agricultural Product Co., Ltd., Fujian, China) were identified by Prof. Zhang Bo, Jilin Agricultural University, Changchun, China, and extracted twice with hot water at 80°C for 2 h. After concentrating the extracts, they were deproteinized with Sevag reagent (chloroform/butanol = 4:1, v/v) and precipitated with 80% alcohol. The resulting polysaccharides were further purified using a diethylaminoethyl cellulose-52 (DEAE-52) anion exchange column (4 × 60 cm; C8930; Solarbio, Beijing, China) and HiPrep™ 26/60 Sephacryl™ S-400 high-resolution (2.6 × 60 cm; 28-9356-05; GE Healthcare, Uppsala, Sweden) and EzLoad 26/60 Chromdex 200 (2.6 × 60 cm; EG007; Bestchrom, Shanghai, China) prep grade columns. The collected polysaccharides (GFPA) were used in subsequent experiments (**Figure S1**).

### 2.2 The physicochemical characterization and structural features of GFPA

#### 2.2.1 Fourier-transform infrared spectroscopy and ultraviolet spectrum

The Fourier-transform infrared (Tianjin Gangdong Technology Development Co., Ltd., Tianjin, China) and ultraviolet (UV) spectra (BioTek, Winooski, VT, USA) of GFPA were determined as previously described (18).

#### 2.2.2 Analysis of the monosaccharide component

A high-performance anion-exchange chromatography system (ICS500; Thermo Fisher Scientific, Waltham, MA, USA) equipped with an electrochemical detector (Dionex ICS 5000 system) and a Dionex™ CarboPac™ PA20 column (150 × 3.0 mm, 10 μm) was used to determine the monosaccharide component of hydrolyzed GFPA. The following conditions were used: column temperature, 30°C; mobile phase A, 0.1 M NaOH; mobile phase B, 0.1 M NaOH and 0.2M NaAc; flow rate, 0.5 mL/min; injection volume, 5 μL and gradient elution (A:B, v/v), 95:5 at 0 min, 80:20 at 30 min, 60:40 at 30.1 min, 60:40 at 45 min, 95:5 at 45.1 min and 95:5 at 60 min (19).

#### 2.2.3 Homogeneity and molecular weight determination

The homogeneity and molecular weight of GFPA were evaluated using a gel chromatography-differential-multi-angle laser light scattering system equipped with the gel-exclusion chromatography columns, Ohpak SB-803, SB-804 and SB-805 in series (300 × 8 mm); a laser light scattering detector (DAWN HELEOS II, Wyatt Technology, Santa Barbara, CA, USA) and a differential detector (Optilab T-rEX, Wyatt Technology). The detection conditions were as follows: column temperature, 45°C; mobile phase, 0.1 M NaNO_3_; flow rate, 0.4 mL/min; injection volume, 100 μL and isocratic elution time, 100 min (20).

#### 2.2.4 Methylation analysis

Methylation analysis was performed by gas chromatography-mass spectrometry, using a previously published method with slight modifications, to determine the bonding structure of GFPA (21). A gas chromatography system (Agilent 7890A; Agilent Technologies Inc., Santa Clara, CA, USA) was used with the following conditions: injection volume, 1 μL; split ratio, 10:1; carrier gas, high purity helium and temperature program set at 140°C for 2 min and increasing to 230°C for 3 min at 3°C/min. A quadrupole mass spectrometry detection system (Agilent 5977B, Agilent Technologies Inc.) equipped with a MassHunter workstation, and an electron impact ion source was used to detect the analyte in full-scan mode with a mass scan range of 30-600 m/z. The characteristic fragments of the methylated polysaccharides were compared to those in an existing database to confirm the glycosidic linkages.

#### 2.2.5 Nuclear magnetic resonance analysis

Purified polysaccharides were lyophilized in D_2_O several times and dissolved in D_2_O (0.5 mL) containing the internal standard trimethylsilylpropanoic acid. One- and two-dimensional nuclear magnetic resonance (NMR) spectra were recorded in Fourier transform mode using an NMR spectrometer (Bruker AVIII 600; Bruker Scientific Technology Co., Ltd., Billerica, MA, USA) equipped with a ^13^C/^1^H dual probe (22).

#### 2.2.6 Scanning electron microscopic analysis

The features of GFPA were recorded by scanning electron microscopy (SEM; Zeiss Merlin Compact; Carl Zeiss AG, Jena, Germany). Lyophilized samples were affixed to a specimen holder with conductive tape and sputtered with gold in a vacuum sputter coater. The samples were then observed at an accelerating voltage of 0.02-30 kV (23).

### 2.3 Animal experiments and GFPA administration

The Animal Ethics Committee of Jilin University approved the animal experimental protocols (SY202103004). Thirty male C57BL/6JGpt mice aged 5 weeks (specific pathogen-free grade, matched for body weight; license number, SCXK [SU] 2018-0008; GemPharmatech Co., Ltd., Jiangsu, China) were maintained at a temperature of 23 ± 1°C and a humidity of 40% to 60%, with a 12-h light/dark cycle and *ad libitum* access to food. Either an HFD (D12492; 60% kcal fat, 20% kcal carbohydrate and 20% kcal protein) or a normal chow diet (NCD; D12450B; 10% kcal fat, 70% kcal carbohydrate and 20% kcal protein; Xiao Shu You Tai Biotechnology Co., Ltd., Beijing, China) was fed to the mice for 10 weeks. The DIO mice were fed an HFD *ad libitum* during the entire experimental period and were randomly divided into four groups (n = 6/group). They were then treated with intragastric administration of 5 mL/kg normal saline (vehicle-treated HFD-fed mice), 3 mg/kg SV (SV-treated HFD-fed mice) or GFPAs at 50 or 100 mg/kg (GFPA-treated HFD-fed mice) once a day for a further 8 weeks. SV, a commercially available statin drug, has use as the positive control drug in many literatures. Therefore, we choose SV as the positive control drug. According to the doses of *G. frondosa* usage in our previous study (15) and the yield of GFPA, the doses of GFPA was finally selected as 50 mg/kg and 100 mg/kg. The NCD-fed (n = 6) mice were intragastrically administered 5 mL/kg normal saline (vehicle-treated NCD-fed mice) for a further 8 weeks. Plasma Glc concentration and body weight were recorded biweekly. On the final day, after fasting for 12 h, blood samples were collected from the caudal vein. Mice were then euthanized by injecting 100 mg/kg sodium pentobarbitone. Tissues, including perirenal white adipose tissue (pWAT), inguinal white adipose tissue (iWAT), epididymal white adipose tissue (eWAT) and liver, were quickly collected for further analysis (**Figure S2**).

### 2.4 Biochemical analyses

As previously described (15), enzyme-linked immunosorbent assay (ELISA) kits (Quanzhou Ruixin Biological Technology Co., Ltd, Fujian, China) were used to determine the concentrations of alanine aminotransferase (ALT, CK203034M), aspartate aminotransferase (AST, CK202596M), total cholesterol (TC, CK-EN20870), triglycerides (TGs, CK-EN21757), high-density lipoprotein cholesterol (HDL-C, 202923M), low-density lipoprotein cholesterol (LDL-C, 202978M), Cer (CK202647M), TLR4 (CK203135M), MyD88 (CK201856M), tumor necrosis factor receptor-associated factor 6 (TRAF6, CK22950), tumor necrosis factor α (TNF-α, CK20852M), interleukin (IL)-1β (CK203063M) and IL-6 (CK203049M) in the sera samples and liver tissues.

### 2.5 Oil red O and hematoxylin and eosin staining

As described in our previous study (24), three types of adipose tissue (eWAT, iWAT and pWAT) and liver tissue specimens were fixed in 4% fixative solution, dehydrated in alcohol, embedded in paraffin and then cut into 5-μm standard sections. The sections were then stained with hematoxylin and eosin (H&E) and observed using an upright optical microscope (Eclipse E100; Nikon, Tokyo, Japan).

Liver sections were incubated in 3.7 mM Oil red O solution at 25°C for 10 min and then incubated in hematoxylin at 25°C for 5 min. The stained sections were coated with malinol and observed under a microscope as previously described (25).

### 2.6 Western blotting

Liver tissues were lysed with radio immunoprecipitation assay buffer (sc-24948; Santa Cruz Biotechnology Inc., Dallas, TX, USA) containing 1% protease inhibitor cocktail (P8340; Sigma-Aldrich, St. Louis, MO, USA) and 2% phenylmethanesulfonyl fluoride (P7626, Sigma-Aldrich). After determining the protein concentration, 40 μg of protein was separated by 10-12% sodium dodecyl sulfate polyacrylamide gel electrophoresis and then transferred onto polyvinylidene fluoride membranes (0.45 μm; GE Healthcare Life Sciences, Marlborough, MA, USA). After blocking with 5% bovine serum albumin at 4°C for 4 h, the membranes were incubated with primary antibodies at 4°C for 12 h and subsequently incubated with the appropriate secondary antibodies at 4°C for 4 h (**Table S1**). The bands were then visualized using an enhanced chemiluminescence kit (PE0010; Solarbio Science & Technology Co., Ltd, Beijing, China) and a Tanon 5200 gel imaging system (Tanon Science & Technology Co., Ltd., Shanghai, China). ImageJ software (National Institutes of Health, Bethesda, MD, USA) was used to quantify the bands.

### 2.7 Statistical analysis

All values are expressed as the mean ± standard error of the mean (S.E.M.). A one-way analysis of variance, followed by a *post-hoc* multiple comparisons (Holm-Sidak) test, was used to analyze the data using DSS Statistics 25 (BONC, Beijing, China). Statistical significance was defined at a *p* value less than 0.05.

## 3 Results

### 3.1 Purification, properties and structural characterization of GFPA

**Figure 1:**
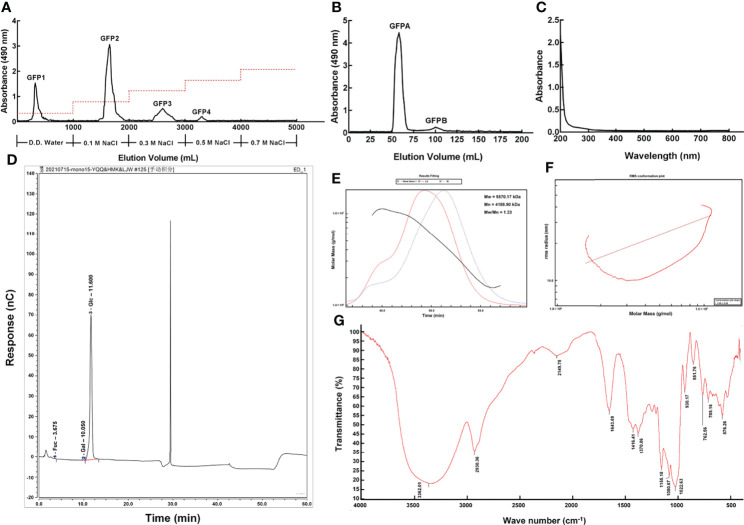
Purification and primary structural characterization of GFPA. GFPA was purified using **(A)** a DEAE-52 anion-exchange column and **(B)** a Chromdex 200 prep grade column. GFPA characterization and glycosyl composition were determined based on **(C)** UV spectra, **(D)** monosaccharide component chromatograms, **(E)** homogeneity and molecular weight, **(F)** molecular configuration analysis and **(G)** Fourier-transform infrared spectroscopy.

Four polysaccharide fractions, named GFP1, GFP2, GFP3 and GFP4, were obtained using a DEAE-52 anion exchange column (**Figure 1A**). GFPA and GFPB fractions were further separated from GFP2 using a Chromdex 200 prep grade column (**Figures 1B** and **S1**). Due to the ultra-low concentration of GFPB, only GFPA was used in subsequent experiments.

Nucleic acid and protein concentrations were low in GFPA, as evidenced by the lack of absorbance peaks at 260 nm and 280 nm by UV spectrophotometry (**Figure 1C**). The monosaccharides in GFPA consisted of Glc, Gal and fucose (Fuc) at a molar ratio of 99.73:0.17:0.10 (**Figure 1D**). The number average molecular weight (Mn) and the weight average molecular weight (Mw) of GFPA were 4,188.90 kDa and 5,570.17 kDa, respectively. A uniform single peak of GFPA was obtained and the polydispersity index (Mw/Mn) of GFPA was 1.33, indicating that it had a relatively homogeneous molecular weight (**Figure 1E**). The root mean square conformation plot (slope: 0.46) showed that the molecular configuration of GFPA was irregular coils, suggesting the existence of branches (**Figure 1F**). The Fourier-transform infrared spectrum (500-4,000 cm^-1^) of GFPA is depicted in **Figure 1G**. A characteristic sugar peak was observed at approximately 3,600-3,200 cm^−1^. A peak at 3,362.01 cm^-1^ corresponded to the O-H stretching vibration, which is a characteristic peak of sugars (26). An absorption peak at 2,930.36 cm^-1^ was attributed to the stretching vibration of C-H (27). The absorption peaks at 1,416.41 cm^-1^ and 1,643.69 cm^-1^ were assigned to C-O and C=O stretching vibrations, respectively. The absorption peak at 1,022.63 cm^-1^ indicated the presence of an O-H variable angle vibration.

GFPA was mainly composed of seven glycosidic fragments (**Table 1** and **Figure S3**); however, Gal and Fuc were not detected due to their ultra-low concentrations. The molar ratios of the non-reducing terminals (t-Glc[p]) and the branching saccharide residues (1,3,4-Glc[p], 1,2,4-Glc[p], 1,3,6-Glc[p] and 1,4,6-Glc[p]) reached 14.524% and 15.017%, respectively, indicating that branched residues may be present (**Table 1**).

**Table 1:** Methylation analysis of GFPA.

Linkages	Methylated glucoside	Molecular weight (Da)	Relative molar ratio (%)	Mass fragments (m/z)
t-Glc[p]	1,5-di-O-acetyl-2,3,4,6-tetra-O-methyl glucitol	323	14.524	59, 71, 87, 102, 118, 129, 145, 161, 162, 205
3-Glc[p]	1,3,5-tri-O-acetyl-2,4,6-tri-O-methyl glucitol	351	3.896	59, 71, 87, 101, 118, 129, 161, 174, 234, 277
4-Glc[p]	1,4,5-tri-O-acetyl-2,3,6-tri-O-methyl glucitol	351	66.563	59, 71, 87, 99, 118, 129, 173, 203, 233
3,4-Glc[p]	1,3,4,5-tetra-O-acetyl-2,6-di-O-methyl glucitol	379	1.475	59, 87, 98, 118, 129, 160, 185, 231, 305
2,4-Glc[p]	1,2,4,5-tetra-O-acetyl-3,6-di-O-methyl glucitol	379	1.226	59, 71, 87, 113, 130, 140, 173, 190, 233, 274
3,6-Glc[p]	1,3,5,6-tetra-O-acetyl-2,4-di-O-methyl glucitol	379	2.222	59, 74, 87, 118, 129, 139, 160, 174, 189, 234, 305
4,6-Glc[p]	1,4,5,6-tetra-O-acetyl-2,3-di-O-methyl glucitol	379	10.094	59, 85, 102, 118, 127, 142, 159, 201, 231, 261, 305

**Figure 2:**
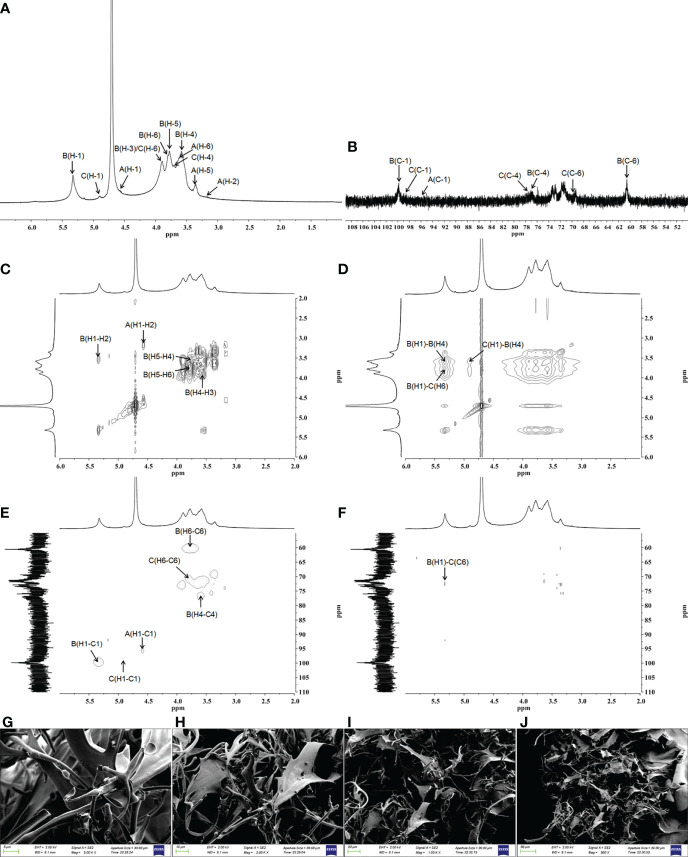
Structural characterization of GFPA *via* NMR and SEM. The structure of GFPA was analyzed by **(A)** ^1^H-NMR, **(B)** ^13^C-NMR, **(C)** COSY, **(D)** NOESY, **(E)** HSQC and **(F)** HMBC. The morphology of GFPA was evaluated by SEM at **(G)** 5,000×, scale bar: 5 μm; **(H)** 2,000×, scale bar: 10 μm; **(I)** 1,000×, scale bar: 20 μm and **(J)** 500×, scale bar: 50 μm.

The detailed structure of GFPA was further confirmed by NMR analysis. The main proton peaks were concentrated in the range of 4.5-5.5 ppm (δ 5.33, 4.90, 4.57) in the ^1^H NMR spectrum. The chemical shift at δ 4.71 ppm was assigned to D_2_O. Other signal peaks were concentrated in the δ 3.1-3.9 region (**Figure 2A**). In the ^13^C NMR spectrum, the nuclear magnetic carbon spectrum signal was mainly concentrated between 55 and 110 ppm, and three anomeric carbon signals were observed, one each at δ 99.83, 98.60 and 95.89 ppm (**Figure 2B**). As shown in **Table 2**, glycosidic bond signals were assigned by combining the HH-correlation spectroscopy (COSY) (**Figure 2C**), nuclear overhauser effect spectroscopy (NOESY, **Figure 2D**), heteronuclear single quantum correlation (HSQC) (**Figure 2E**) and heteronuclear multiple bond correlation (HMBC) (**Figure 2F**) data, and related references (28–30).

**Table 2:** Summary of ^1^H and ^13^C NMR chemical shifts for GFPA.

Glycosyl residues	H1/C1	H2/C2	H3/C3	H4/C4	H5/C5	H6a,b/C6	H6b
β-D-Glcp-(1→ (Residue A)	4.57	3.18	3.67	3.31	3.37	3.65	3.62
	95.89	74.02	76.15	69.60	75.95	60.21	
→4)-α-D-Glcp-(1→ (Residue B)	5.33	3.54	3.89	3.58	3.78	3.80	3.70
	99.83	71.61	73.17	76.91	71.31	60.56	
→4,6)-β-D-Glcp-(1→ (Residue C)	4.90	3.49	3.64	3.71	3.74	3.89	3.80
	98.60	72.58	74.27	77.42	72.42	70.14	

Using the NOESY atlas (**Figure 2D**), the anomeric hydrogen of the glycosidic bond →4)-α-D-Glcp(1→ was found to have signal peaks related to its own H4, showing the following link mode: →4)-α-D-Glcp(1→4)-α-D-Glcp(1→. The anomeric hydrogens of →4)-α-D-Glcp(1→ and H6 of →4,6)-β-D-Glcp(1→ had related signal peaks, indicating the existence of →4)-α-D-Glcp(1→4,6)-β-D-Glcp(1→. The anomeric hydrogen of →4,6)-β-D-Glcp(1→ and H4 of →4)-α-D-Glcp(1→ had related signal peaks, indicating the existence of →4,6)-β-D-Glcp(1→4)-α-D-Glcp(1→.

In the HMBC spectrum (**Figure 2F**), the anomeric hydrogen of the glycosidic bond, →4)-α-D-Glcp(1→, and C6 of →4,6)-β-D-Glcp(1→ had related signal peaks, indicating the existence of →4)-α-D-Glcp(1→4,6)-β-D-Glcp(1→.

The microstructure and morphological characteristics of GFPA were analyzed by SEM. In the aggregated state, an irregular branch network structure with an uneven surface was noted, suggesting an amorphous structure of GFPA. The surface of GFPA was uneven in thickness and showed crests and sags, which may have been caused by the branched structure of GFPA (**Figures 2G–J**) (31).

### 3.2 GFPA exhibited hypolipidemic effects

**Figure 3:**
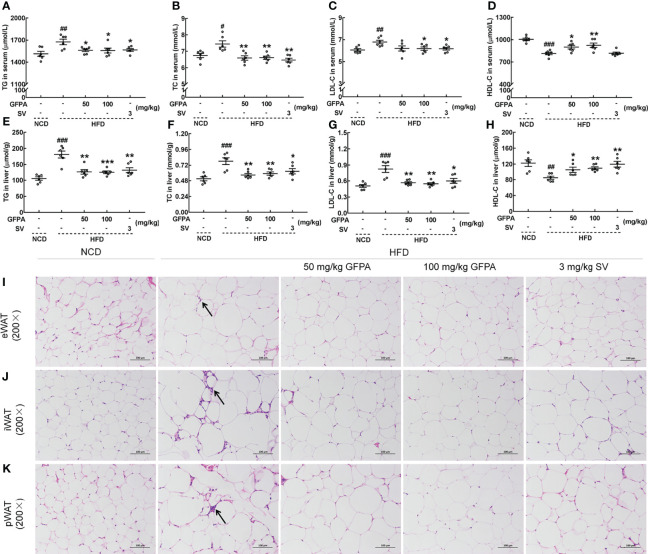
GFPA suppressed hyperlipidemia in mice with DIO. Eight weeks of GFPA administration decreased the concentrations of **(A)** TGs, **(B)** TC and **(C)** LDL-C and increased the concentration of **(D)** HDL-C in the sera of HFD-fed mice. Eight weeks of GFPA administration decreased the concentrations of **(E)** TGs, **(F)** TC and **(G)** LDL-C and increased the concentration of **(H)** HDL-C in the livers of HFD-fed mice. Data are expressed as means ± S.E.M. (n = 6). ^#^ *p* < 0.05, ^##^ *p* < 0.01 and ^###^ *p* < 0.001 versus NCD-fed mice; *p < 0.05, **p < 0.01 and ***p < 0.001 versus HFD-fed mice. Pathological alterations in **(I)** eWAT, **(J)** iWAT and **(K)** pWAT were evaluated by H&E staining (magnification: 200×; scale bar: 50 μm). The black arrow indicates the inflammatory infiltration of lymphocytes in HFD-fed mice.

Significant increases in body weight and plasma Glc concentration (*p* < 0.001, **Table S2**) were noted in HFD-fed mice, and these effects were suppressed by 8 weeks of GFPA administration (*p* < 0.05) (**Table S2**). After GFPA treatment, decreased serum TGs (*p* < 0.05) (**Figure 3A**), TC (*p* < 0.01) (**Figure 3B**) and LDL-C levels (100 mg/kg GFPA, *p* < 0.05) (**Figure 3C**), and increased serum HDL-C levels (50 mg/kg GFPA, *p* < 0.05; 100 mg/kg GFPA, *p* < 0.01) (**Figure 3D**) in HFD-fed mice were observed. The high concentrations of TGs, TC and LDL-C (*p* < 0.001) and the low concentration of HDL-C (*p* < 0.01) in the liver of HFD-fed mice were noted, but these were regulated by over 29.7% (50 mg/kg GFPA, *p* < 0.01; 100 mg/kg GFPA, *p* < 0.001) (**Figure 3E**), over 24.1% (*p* < 0.01) (**Figure 3F**), over 31.5% (*p* < 0.01) (**Figure 3G**) and over 24.9% (50 mg/kg GFPA, *p* < 0.05; 100 mg/kg GFPA, *p* < 0.01) (**Figure 3H**), respectively, after GFPA administration. Comparatively, SV showed beneficial effects on all of the aforementioned lipid parameters (*p* < 0.05) except serum HDL-C concentration (**Figures 3A–H**). Larger adipocytes and increased inflammatory infiltration of lymphocytes were noted in the three WATs of HFD-fed mice relative to NCD-fed mice. These effects were ameliorated after GFPA and SV administration (**Figures 3I–K**).

### 3.3 GFPA alleviated hepatic steatosis

**Figure 4:**
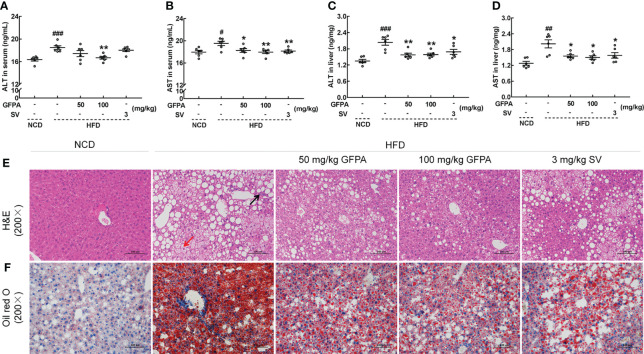
GFPA alleviated hepatic steatosis in mice with DIO. In HFD-fed mice, GFPA suppressed the serum concentrations of **(A)** ALT and **(B)** AST and the hepatic concentrations of **(C)** ALT and **(D)** AST. Data are expressed as means ± S.E.M. (n = 6). ^#^ *p* < 0.05, ^##^ *p* < 0.01 and ^###^ *p* < 0.001 versus NCD-fed mice; *p < 0.05 and **p < 0.01 versus HFD-fed mice. Pathological alterations in the liver were evaluated by **(E)** H&E staining (magnification: 200×; scale bar: 50 μm) and **(F)** Oil red O staining (magnification: 200×; scale bar: 50 μm). The black arrow indicates the inflammatory infiltration of lymphocytes in HFD-fed mice, and the red arrow indicates ballooning degeneration, cytoplasmic vacuolation and cellular swelling in HFD-fed mice.

Significant increases in ALT (*p* < 0.001) (**Figures 4A, C**) and AST (*p* < 0.05) (**Figures 4B, D**) concentrations were noted in the sera and livers of HFD-fed mice. After 8 weeks of GFPA treatment, the ALT concentration decreased by more than 9.6% in the sera (100 mg/kg GFPA, *p* < 0.01) (**Figure 4A**) and by more than 22.1% in the liver (*p* < 0.01) (**Figure 4C**). After the same treatment period, the AST concentration decreased by more than 6.5% in the sera (50 mg/kg GFPA, *p* < 0.05; 100 mg/kg GFPA, *p* < 0.01) (**Figure 4B**) and by more than 23.1% in the liver (*p* < 0.05) (**Figure 4D**). SV treatment also significantly decreased the AST concentration in the sera (*p* < 0.01) (**Figure 4B**) and the ALT (*p* < 0.05) (**Figure 4C**) and AST (*p* < 0.05) (**Figure 4D**) concentrations in the liver. In the livers of HFD-fed mice, typical pathological characteristics including hepatocyte steatosis, cytoplasmic vacuolation and lymphocytic infiltration were noted (**Figure 4E**). However, GFPA and SV alleviated fat accumulation and inflammatory infiltration, exhibiting significant protective effects in the liver (**Figure 4E**). Based on Oil red O staining results, GFPA and SV reduced the amount of lipids in HFD-fed mice (**Figure 4F**).

### 3.4 GFPA ameliorated metabolic inflammation by inhibiting TLR4/NF-κB signaling

**Figure 5:**
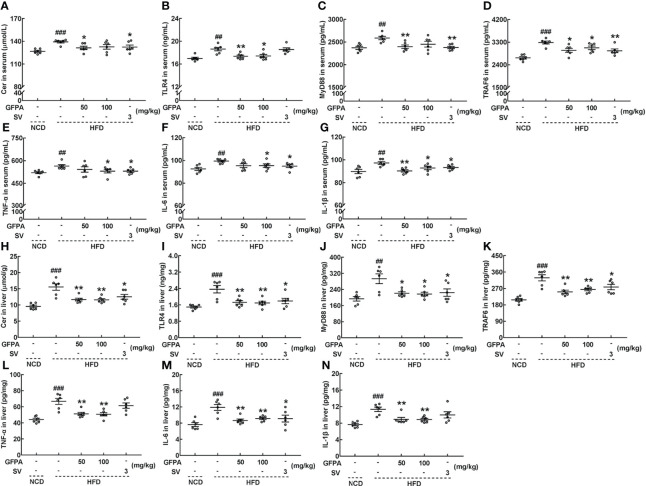
GFPA suppressed the obesity-induced inflammatory response in mice with DIO. GFPA decreased the serum concentrations of **(A)** Cer, **(B)** TLR4, **(C)** MyD88, **(D)** TRAF6, **(E)** TNF-α, **(F)** IL-6 and **(G)** IL-1β and the hepatic concentrations of **(H)** Cer, **(I)** TLR4, **(J)** MyD88, **(K)** TRAF6, **(L)** TNF-α, **(M)** IL-6 and **(N)** IL-1β in HFD-fed mice. Data are expressed as means ± S.E.M. (n = 6). ^##^ *p* < 0.01 and ^###^ *p* < 0.001 versus NCD-fed mice; *p < 0.05 and **p < 0.01 versus HFD-fed mice.

The concentrations of related inflammatory factors were determined using our previously published method (15). In HFD-fed mice, after 8 weeks of GFPA administration, the serum concentrations of Cer (50 mg/kg GFPA, *p* < 0.05) (**Figure 5A**), TLR4 (50 mg/kg GFPA, *p* < 0.01; 100 mg/kg GFPA, *p* < 0.05) (**Figure 5B**), MyD88 (50 mg/kg GFPA, *p* < 0.01) (**Figure 5C**), TRAF6 (*p* < 0.05) (**Figure 5D**), TNF-α (100 mg/kg GFPA, *p* < 0.05) (**Figure 5E**), IL-6 (100 mg/kg GFPA, *p* < 0.05) (**Figure 5F**) and IL-1β (50 mg/kg GFPA, *p* < 0.01; 100 mg/kg GFPA, *p* < 0.05) (**Figure 5G**) were all decreased, and the hepatic concentrations of Cer (*p* < 0.01) (**Figure 5H**), TLR4 (*p* < 0.01) (**Figure 5I**), MyD88 (*p* < 0.05) (**Figure 5J**), TRAF6 (*p* < 0.01) (**Figure 5K**), TNF-α (*p* < 0.01) (**Figure 5L**), IL-6 (*p* < 0.01) (**Figure 5M**) and IL-1β (*p* < 0.01) (**Figure 5N**) were all decreased. In contrast, SV exhibited beneficial effects on the concentrations of all of these inflammatory factors (*p* < 0.05) except serum TLR4 concentration and hepatic TNF-α and IL-1β concentrations (**Figures 5A–N**).

**Figure 6:**
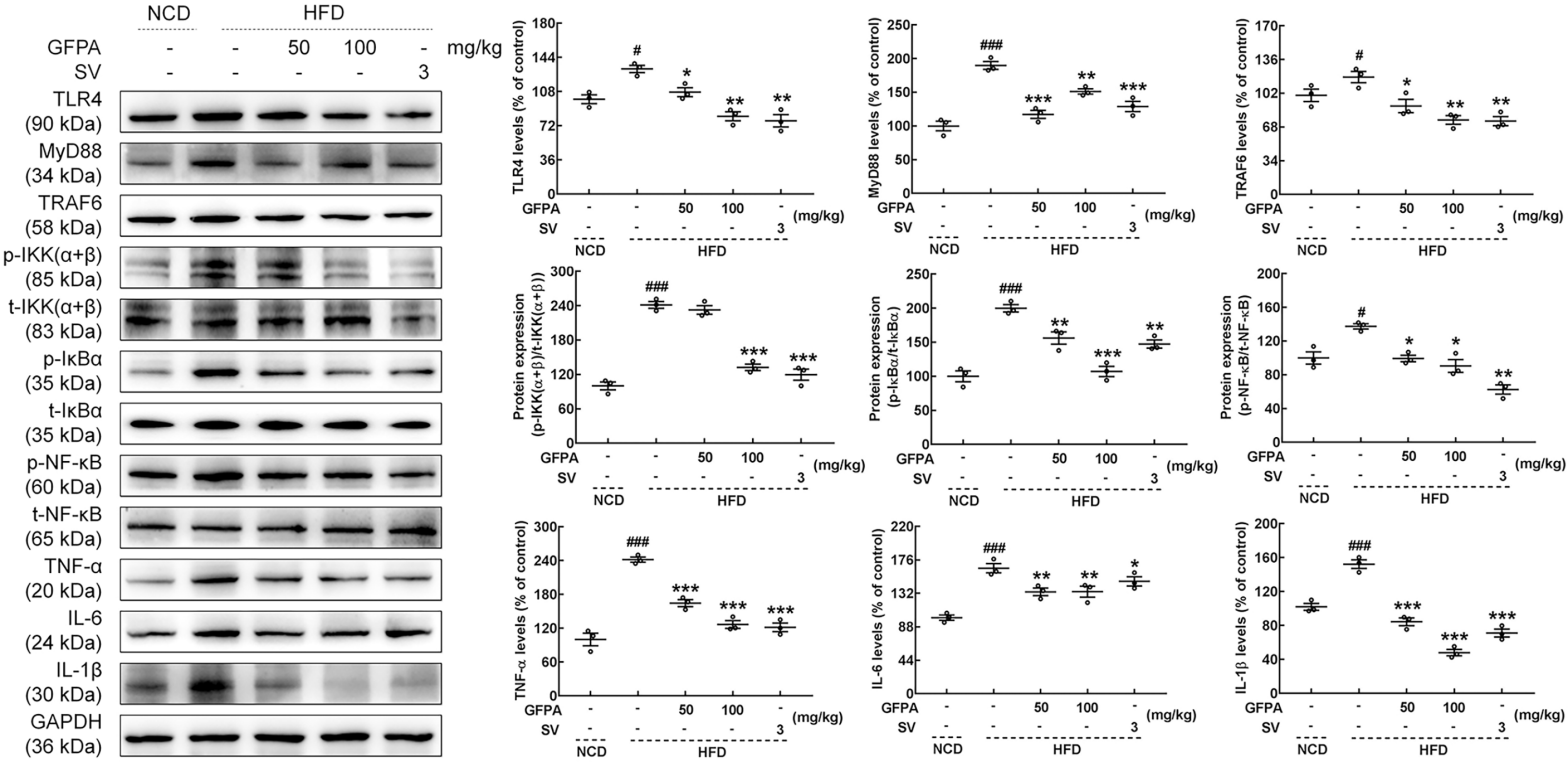
GFPA attenuated metaflammation by regulating TLR4/NF-κB signaling in the livers of mice with DIO. GFPA suppressed the expression levels of TLR4, MyD88, TRAF6, TNF-α, IL-6 and IL-1β and the phosphorylation levels of IKK(α+β), IκBα and NF-κB in the livers of HFD-fed mice. Quantification data were normalized to glyceraldehyde-3-phosphate dehydrogenase (GAPDH) or the corresponding total protein concentration. Data are expressed as means ± S.E.M. (n = 3). ^#^ *p* < 0.05 and ^###^ *p* < 0.001 versus NCD-fed mice; *p < 0.05, **p < 0.01 and ***p < 0.001 versus HFD-fed mice.

TLR4/NF-κB signaling plays important roles during *G. frondosa*-mediated lipid metabolism (15). Hence, the effects of GFPA on TLR4/NF-κB signaling were investigated. Similar to the effects of SV treatment, the expression levels of TLR4 (50 mg/kg GFPA, *p* < 0.05; 100 mg/kg GFPA, *p* < 0.01), MyD88 (50 mg/kg GFPA, *p* < 0.001; 100 mg/kg GFPA, *p* < 0.01), TRAF6 (50 mg/kg GFPA, *p* < 0.05; 100 mg/kg GFPA, *p* < 0.01), phosphorylated (p)-inhibitor of nuclear factor kappa-B kinase (IKK) (α+β) (100 mg/kg GFPA, *p* < 0.001), p-inhibitor of nuclear factor kappa-B α (p-IκBα, 50 mg/kg GFPA, *p* < 0.01; 100 mg/kg GFPA, *p* < 0.001), p-NF-κB (*p* < 0.05), TNF-α (*p* < 0.001), IL-6 (*p* < 0.01) and IL-1β (*p* < 0.001) in the livers of HFD-fed mice were significantly downregulated by GFPA treatment (**Figure 6**).

## 4 Discussion

Here, we report that the polysaccharide GFPA, which was purified from *G. frondosa* fruiting bodies, had an Mn of 4,188.90 kDa, showed good uniformity and had anti-obesity effects. In a previous study, a water-soluble polysaccharide named GFP30-2-a, with a molecular weight of 2,040 kDa, a (1→4)-α-D-Glcp backbone and a small number of terminal β-D-Galp residues, was isolated from *G. frondosa* (29). Another crude polysaccharide preparation obtained from *G. frondosa* has inhibitory effects on weight gain and reverses liver damage in rats (32). We found that GFPA mainly consisted of →4)-α-D-Glcp-(1→, β-D-Glcp-(1→ and →4,6)-β-D-Glcp-(1→, and we confirmed the existence of a branched structure, which may be highly related to its anti-obesity effects.

In our study, male mice with DIO were used to observe the effects of GFPA on obesity, because the female estrous cycle may induce undesirable experimental variability. Moreover, gonadal hormones can influence the distribution of fat and the development of obesity (33). According to our results, GFPA had greater effects than SV in reducing body weight and blood glucose of HFD-fed mice, in other aspects, GFPA had similar effects with SV. GFPA inhibited body weight gain in HFD-fed mice and decreased the size of adipocytes in three types of WAT, thus confirming its anti-obesity effects. GFPA modulated hypolipidemic factors and alleviated hepatic fat accumulation in HFD-fed mice, indicating its ability to regulate lipid metabolism. GFPA also ameliorated the inflammatory infiltration of lymphocytes in the three WATs and in the liver and decreased the concentrations of pro-inflammatory factors (TNF-α, IL-6 and IL-1β) in HFD-fed mice, which further confirmed its anti-inflammatory effects.

The relationship between lipid dysregulation and obesity-induced inflammation is bidirectional (34). TNF-α and IL-6 induce adipogenesis and increase the production and secretion of TGs, which promote the development of hyperlipidemia and fatty liver disease (35). Excessive storage of TGs induces the secretion of pro-inflammatory adipokines, including TNF-α, IL-6 and IL-1β, which causes systemic metabolic inflammation (36). Chronic inflammation is defined as chronic low-grade inflammation established by metabolic and inflammatory cells to defend against an overload of energetic nutrients in obese patients. It is a hallmark of obesity (3). Accordingly, the progression of chronic inflammation largely depends on pro-inflammatory cytokines produced by genes under the transcriptional control of NF-κB (37). We showed that GFPA exhibited anti-obesity effects by inhibiting chronic inflammation in HFD-fed mice.

TLR4 signaling initiates a cascade of events that cause NF-κB activation (38), and it has been identified as a trigger of obesity-associated chronic inflammation (39). In brief, activated TLR4 activates MyD88, which then activates a signalosome composing of TRAF6 and TAK, leading to the activation of TAK1 by autophosphorylation. Phosphorylated TAK1 then causes the activation of IKK (40). We found that GFPA decreased the concentrations of TLR4, MyD88 and TRAF6 in the serum and liver, as determined by ELISA, and reduced the expression levels of TLR4, MyD88 and TRAF6 in the liver, as determined by western blotting. IKK phosphorylates the NF-κB inhibitor IκBα and marks it for degradation. NF-κB is subsequently released, after which it translocates to the nucleus to regulate the transcription of pro-inflammatory cytokines (41, 42). Pro-inflammatory cytokines also increase the *de novo* synthesis of Cer and exacerbate inflammation (43). GFPA decreased the levels of p-IKK(α+β), p-IκBα and p-NF-κB in the livers of HFD-fed mice, suggesting that it suppressed chronic inflammation by inhibiting TLR4/NF-κB signaling.

Cer is a component of lipoproteins, total plasma Cer was higher in obese individuals compared with lean participants, hepatic Cer secretion is increased in rodent obesity and that the liver is the primary source of circulating Cer (44). Cer and chronic inflammation are inextricably linked. Increased Cer concentrations are observed in various tissues (liver, muscle and hypothalamus) of rodents and humans with obesity. Therefore, decreased circulating Cer concentrations may be responsible for reducing fat mass (45). Cer is reported to be a molecular intermediate in the transmission of inflammatory signals (44). Cer activates proinflammatory pathways *via* amplification of TLR4-mediated inflammation (44). The activation of TLR4 increases the synthesis of Cer and its concentration in plasma lipoproteins (46). Cytokines induced by TLR4, such as TNF-α, also increase cellular Cer concentrations (47). Furthermore, Cer increases IKK signaling, as assessed by IκBα degradation, and promotes the activation of NF-κB, and consequently, inhibiting Cer synthesis suppresses the production of pro-inflammatory factors (44). We found that GFPA significantly decreased the concentration of Cer in the sera and livers of HFD-fed mice, which is consistent with our previous findings (15). These findings confirm that the inhibition of chronic inflammation by GFPA plays an important role in its anti-obesity effects (**Figure S4**).

This study had some limitations that should be noted. First, the structure-activity relationships between *G. frondosa* polysaccharides and their anti-obesity effects require further study. Second, the anti-obesity effects of GFPA were only demonstrated in HFD-fed mice. Therefore, numerous additional studies are required prior to the clinical application of GFPA. Furthermore, a high degree of branching was observed in the structural analysis of GFPA, and a previous study reported the existence of triple-helix structures in *G. frondosa* polysaccharides (48). Hence, the relationship between the three-dimensional structure and the anti-obesity effects of *G. frondosa* polysaccharides requires further research. This will facilitate the design of functional foods and health-promoting pharmaceuticals based on chemical modifications.

## 5 Conclusions

GFPA, a purified polysaccharide from *G. frondosa*, was systemically structurally characterized. In HFD-fed mice, GFPA alleviated hepatic steatosis and the inflammatory response and exhibited strong anti-obesity effects. These effects were shown to be related to the suppression of chronic inflammation through TLR4/NF-κB signaling. These data support the application of GFPA as an agent targeting chronic inflammation for the treatment of obesity.

## Data availability statement

The original contributions presented in the study are included in the article/**Supplementary Material**. Further inquiries can be directed to the corresponding authors.

## Ethics statement

The animal study was reviewed and approved by the animal ethics committee of Jilin University (SY202103004).

## Author contributions

DW designed the experiments. XJ, JH, YFZ and ZJL performed the experiments. XJ processed the data. XJ wrote the paper. DW, LRT, LZL, YLZ and YL revised the paper. All authors contributed to the article and approved the submitted version.

## Funding

This work was supported by the Science and Technology Development Project in Jilin Province, China (20200708068YY and 20210401169YY), the National Key Project of “Science and Technology for Economy 2020” (2020YFF0414043), the Science and Technology Research Project from Education Department of Jilin Province in China (JJKH20211227KJ), and the China Agriculture Research System of MOF and MARA (CARS-20-08B).

## Acknowledgments

We thank Prof. Zhang Bo from the Jilin Agricultural University for the fungus appraisal.

## Conflict of interest

The authors declare that the research was conducted in the absence of any commercial or financial relationships that could be construed as a potential conflict of interest.

## Publisher’s note

All claims expressed in this article are solely those of the authors and do not necessarily represent those of their affiliated organizations, or those of the publisher, the editors and the reviewers. Any product that may be evaluated in this article, or claim that may be made by its manufacturer, is not guaranteed or endorsed by the publisher.

## Abbreviations

ALT, Alanine aminotransferase; AST, Aspartate aminotransferase; Cer, Ceramide; COSY, Correlation spectroscopy; DEAE-52, Diethylaminoethyl cellulose-52; DIO, Diet-induced obesity; ELISA, Enzyme-linked immunosorbent assay; eWAT, Epididymal white adipose tissue ; Fuc, Fucose; Gal, Galactose; G. frondosa, Grifola frondosa; GFPA, Polysaccharides purified from Grifola frondosa; Glc, Glucose; H&E, Hematoxylin and eosin; HDL-C, High-density lipoprotein cholesterol; HFD, High-fat diet; HMBC ,Heteronuclear multiple bond correlation; HSQC, Heteronuclear single quantum correlation; IκBα, Inhibitor of nuclear factor kappa-B α; IKK(α+β), Inhibitor of nuclear factor kappa-B kinase (α+β); IL, Interleukin; iWAT, Inguinal white adipose tissue; LDL-C, Low-density lipoprotein cholesterol; Mn, Number average molecular weight; Mw, Weight average molecular weight; MyD88, Myeloid differential protein-88; NCD, Normal chow diet; NF-κB, Nuclear factor kappa-B; NOESY, Nuclear overhauser effect spectroscopy; Phosphorylated-, P-; pWAT, Perirenal white adipose tissue; SEM, Scanning electron microscopy; S.E.M., Standard error of the mean; SV, Simvastatin; TC, Total cholesterol; TGs, Triglycerides; TLR4, Toll-like receptor 4; TNF-α, Tumor necrosis factor α; TRAF6, Tumor necrosis factor receptor-associated factor 6; UV, Ultraviolet.
