# Supplementary material for: The anti-obesity effects of a water-soluble glucan from Grifola frondosa via the modulation of chronic inflammation
Source: Front Immunol. 2022 Jul 28;13:962341. doi: 10.3389/fimmu.2022.962341 (PMC9367694; doi:10.3389/fimmu.2022.962341)
Supplement: Supplementary file 1 [file DataSheet_1.docx]

Supplementary Material

**Supplementary Table S1** Primary antibodies used for western blotting.

| Name | Item number | Molecular Weight (kDa) | Dilution rate |
| --- | --- | --- | --- |
| TLR4 *^a^* | ab217274 | 90 | 1/300 |
| MyD88 *^b^* | bs-1047R | 34 | 1/2000 |
| TRAF6 *^a^* | ab33915 | 58 | 1/2000 |
| p-IKK(α+β) *^b^* | bs-3236R | 85 | 1/1000 |
| t-IKK(α+β) *^b^* | bs-10123R | 83 | 1/1000 |
| p-IκBα *^a^* | ab133462 | 35 | 1/2000 |
| t-IκBα *^c^* | A19714 | 35 | 1/1000 |
| p-NF-κB *^a^* | ab28856 | 60 | 1/1000 |
| t-NF-κB *^a^* | ab32536 | 65 | 1/2000 |
| TNF-α *^d^* | 3707S | 20 | 1/1000 |
| IL-6 *^d^* | 12912S | 24 | 1/1000 |
| IL-1β *^c^* | A1112 | 30 | 1/1000 |
| GAPDH *^a^* | ab181602 | 36 | 1/500 |

Antibodies were purchased from *^a^*Abcam (Shanghai, China), *^b^*Bioss (Beijing, China), *^c^*Abclonal (Wuhan, China) and *^d^*Cell Signaling Technology, Inc. (Shanghai, China).

**Supplementary Table S2** The effects of GFPA on body weight and plasma glucose concentration.

|  | week | NCD | HFD | HFD+50 mg/kg GFPA | HFD+100 mg/kg GFPA | HFD+3 mg/kg SV |
| --- | --- | --- | --- | --- | --- | --- |
| Body weight (g) | 0 | 27.98±0.15 | 37.38±0.51^###^ | 37.05±0.58 | 37.35±0.37 | 37.57±0.55 |
|  | 2 | 28.38±0.12 | 43.38±0.95^###^ | 36.30±0.60^***^ | 35.40±0.75^***^ | 38.48±0.55^**^ |
|  | 4 | 29.23±0.13 | 46.93±0.59^###^ | 40.33±0.63^***^ | 38.90±0.84^***^ | 40.95±0.50^***^ |
|  | 6 | 29.90±0.17 | 49.92±0.27^###^ | 43.53±0.76^***^ | 42.28±0.74^***^ | 44.75±0.48^***^ |
|  | 8 | 30.20±0.25 | 51.58±0.06^###^ | 47.47±0.65^***^ | 44.45±0.90^***^ | 47.42±0.55^***^ |
| Plasma glucose  (mmol/L) | 0 | 8.2±0.2 | 11.1±0.5^###^ | 11.1±0.6 | 11.3±0.8 | 11.0±0.5 |
|  | 2 | 8.3±0.1 | 12.4±0.4^###^ | 11.3±0.3 | 10.6±0.7^*^ | 11.1±0.2^*^ |
|  | 4 | 8.7±0.4 | 12.5±0.5^###^ | 11.4±0.8 | 10.3±0.8^*^ | 11.0±0.2^*^ |
|  | 6 | 8.8±0.3 | 12.7±0.3^###^ | 10.1±0.4^***^ | 9.9±0.3^***^ | 10.8±0.6^*^ |
|  | 8 | 8.9±0.1 | 12.9±0.7^###^ | 10.8±0.2^*^ | 10.4±0.4^*^ | 10.9±0.1^*^ |

Data are expressed as means ± S.E.M. (n = 6). ^###^ *p* < 0.001 versus NCD-fed mice; * *p* < 0.05, ** *p* < 0.01 and *** *p* < 0.001 versus HFD-fed mice.


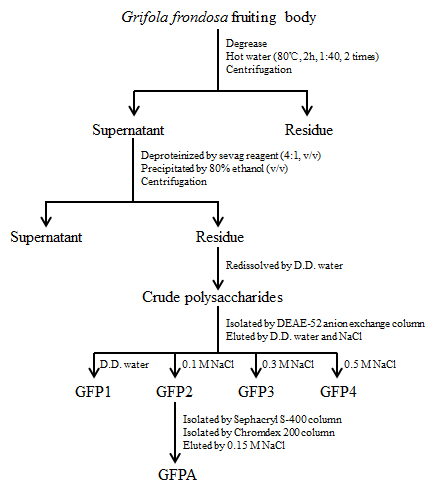


**Supplementary Figure S1** Purification of polysaccharides isolated from *Grifola frondosa* fruiting bodies.


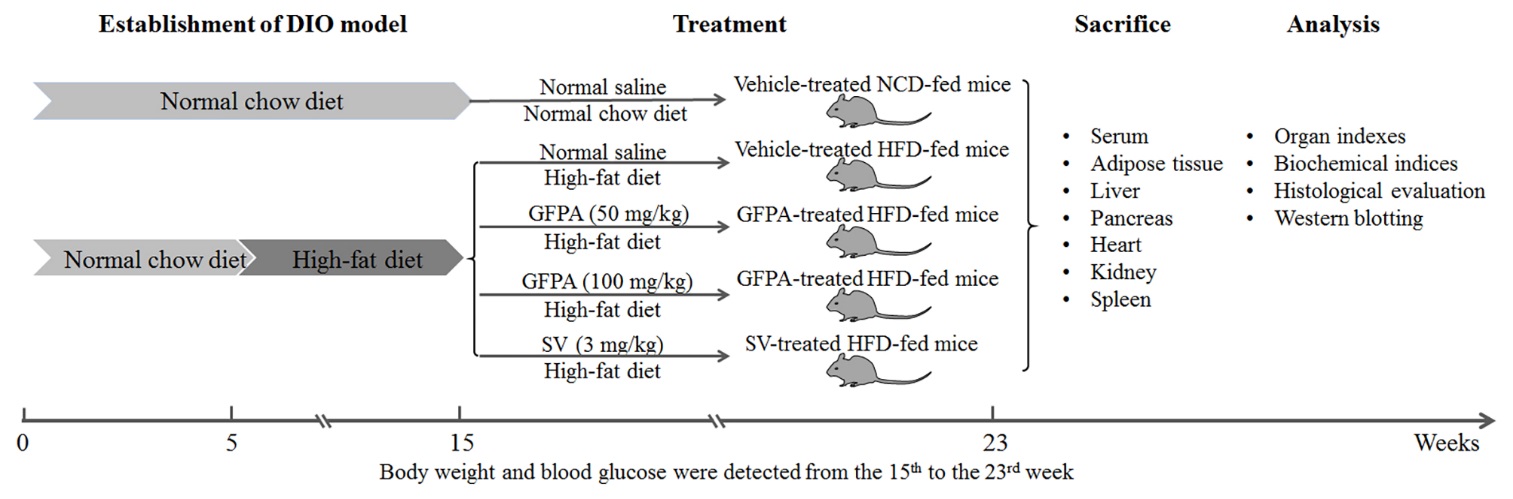


**Supplementary Figure S2** The process of establishing DIO mice and drug administration.

**
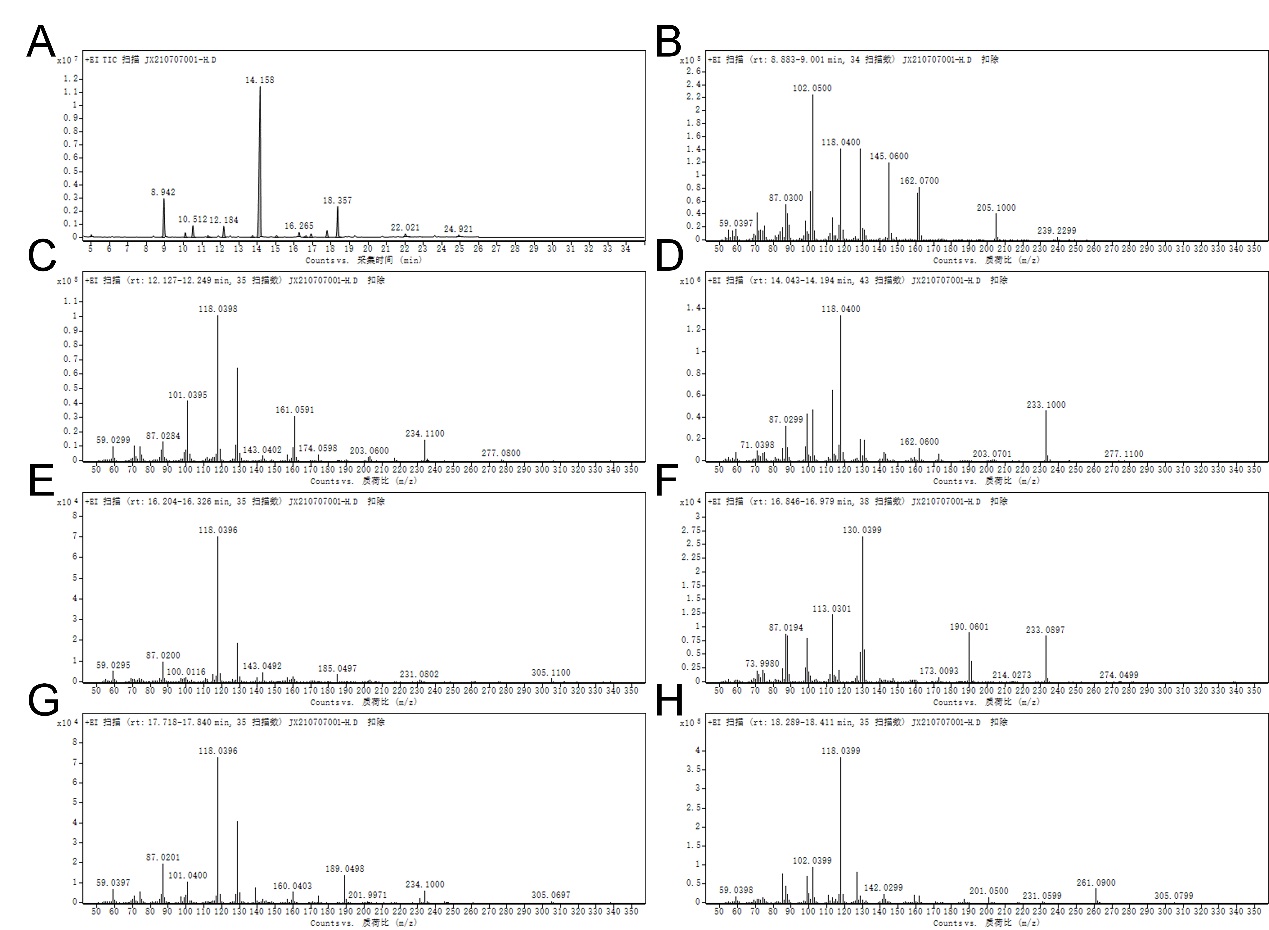
**

**Supplementary Figure S3** (A) The total ion current in GC/MS. The ion fragments of different linkage patterns in EI/MS. (B) t-Glc[p], (C) 3-Glc[p], (D) 4-Glc[p], (E) 3,4-Glc[p], (F) 2,4-Glc[p], (G) 3,6-Glc[p] and (H) 4,6-Glc[p].


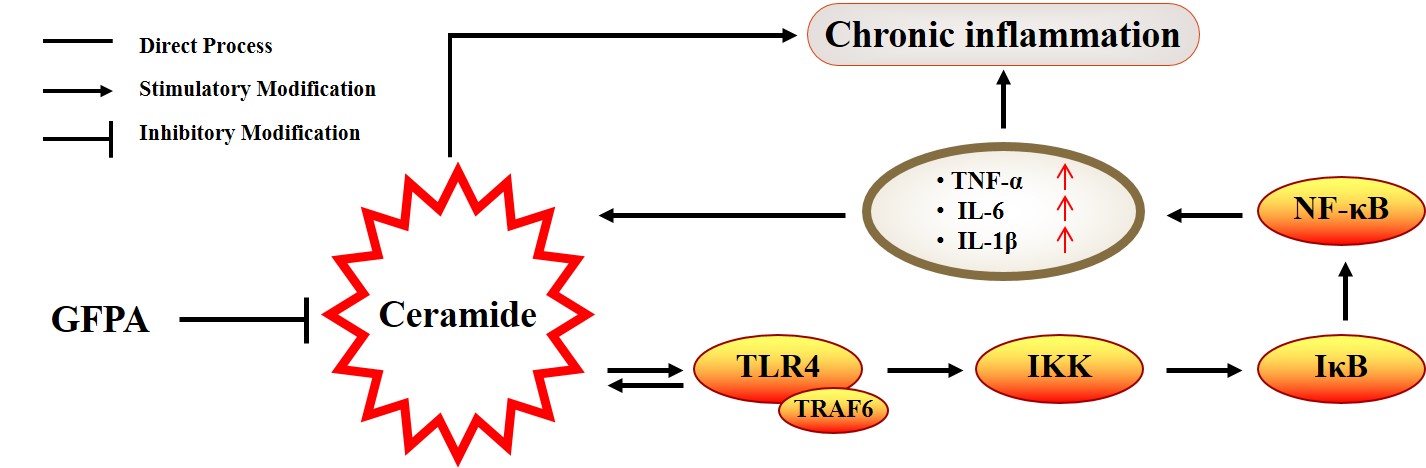


**Supplementary Figure S4** The schematic diagram of mechanisms involved in the anti-inflammtory effects of GFPA in DIO mice.
